# Scalable representations of diseases in biomedical ontologies

**DOI:** 10.1186/2041-1480-2-S2-S6

**Published:** 2011-05-17

**Authors:** Stefan Schulz, Kent Spackman, Andrew James, Cristian Cocos, Martin Boeker

**Affiliations:** 1 Institute for Medical Informatics, Statistics and Documentation, Medical University of Graz, Austria; 2 International Health Terminology Standards Development Organisation, Copenhagen, Denmark; 3 Department of Paediatrics, University of Toronto, Toronto, Ontario, Canada; 4 Saint Francis Xavier University, Antigonish, Nova Scotia, Canada; 5 Institute of Medical Biometry und Medical Informatics, University Medical Center Freiburg, Germany

## Abstract

**Background:**

The realm of pathological entities can be subdivided into pathological dispositions, pathological processes, and pathological structures. The latter are the bearer of dispositions, which can then be realized by their manifestations — pathologic processes. Despite its ontological soundness, implementing this model via purpose-oriented domain ontologies will likely require considerable effort, both in ontology construction and maintenance, which constitutes a considerable problem for SNOMED CT, presently the largest biomedical ontology.

**Results:**

We describe an ontology design pattern which allows ontologists to make assertions that blur the distinctions between dispositions, processes, and structures until necessary. Based on the domain upper-level ontology BioTop, it permits ascriptions of location and participation in the definition of pathological phenomena even without an ontological commitment to a distinction between these three categories. An analysis of SNOMED CT revealed that numerous classes in the findings/disease hierarchy are ambiguous with respect to process vs. disposition. Here our proposed approach can easily be applied to create unambiguous classes. No ambiguities could be defined regarding the distinction of structure and non-structure classes, but here we have found problematic duplications.

**Conclusions:**

We defend a judicious use of disjunctive, and therefore ambiguous, classes in biomedical ontologies during the process of ontology construction and in the practice of ontology application. The use of these classes is permitted to span across several top-level categories, provided it contributes to ontology simplification and supports the intended reasoning scenarios.

## Introduction

Clinical medicine, public health, and biomedical research focus on diseases with regard to their etiology, manifestations, diagnostic and therapeutic aspects. Although disease clearly constitutes the central organizational tenet in medicine, a principled ontological analysis of the characteristics of disease entities exhibits major difficulties. The delineation between health and disease is not only intrinsically vague, but also depends on medical, legal, and cultural criteria [1]. One controversial issue among many, for instance, is the phenomenon of ageing — to what extent do signs and symptoms of an ageing organism constitute a normal phenomenon, typical for the end phase of human life, or to what extent do they indicate diseases requiring therapeutic actions?

While the boundary, or better the continuum [2], between the normal and the abnormal is intrinsically problematic, there are additional reasons for ontology engineers to remain agnostic with regard to this issue and to avoid a simplistic bipartition. An equally important challenge for representing medically relevant phenomena – the complex of diseases, disorders, and illnesses in a broader sense — is to find the right upper-level categories under which representational units, known as "nodes" in an ontology, are to be appropriately placed.

Whereas a major *raison d'être* of formal ontologies is to promote an unambiguous and commonly agreed typology for categorizing the entities of the domain to be represented, the representation of diseases in biomedical ontologies is widely idiosyncratic. Current biomedical ontologies and terminology systems subscribe to diverging and partly contradicting concepts regarding diseases. Diseases have often been categorized as processes, events, or states [1]; a recent proposal regards diseases, first of all, as dispositional entities [3]. This approach has been further refined by the Ontology of General Medical Science (OGMS) [4], with the consideration of disorders as the abnormal bodily components and disease processes as the manifestations of diseases (dispositions).

As much as this approach is theoretically well elaborated, it may encounter resistance when ontology engineering and maintenance are guided by pragmatic, purpose-oriented principles and equipped with limited resources. There are numerous cases in which a conflation of pathological disposition, process, and structure is perfectly acceptable. In other cases the distinction may be relevant for some diseases but not for others. Generally, in incremental ontology design processes, Scheuermann's distinction is not a priority in their initial stages and ontology engineers may want to leave related modeling decisions open until concrete needs arise.

The purpose of this paper is to describe an ontology engineering approach toward disease that is characterized by intuitiveness, user-friendliness, ontological soundness, computability, and scalability. As a concrete use case we analyze how the proposed approach can be sensibly implemented in the current ontological redesign activities of SNOMED CT [5].

## Background

Although our approach is based upon the work by Williams [3] and Scheuermann [4], we do not follow their proposed terminology and do not subscribe to the alleged ontological distinction between entities linguistically characterized by terms including the terms "disease" and "disorder". Our main concern is that the meaning Williams and Scheuermann give these terms does not correspond to the usage of these terms by practitioners, researchers or terminologists. We base our arguments on related work as well as on empirical data.

Much medical literature, and theoretic philosophical deliberations towards disease and health such as by Murphy [6], simply use "disease" and "disorder" interchangeably. Where authors make a conceptual distinction, disease is often conceived as a refinement of disorder. According to Hoffman [7] disease is the explanation of disorders whereas illness/disorder, conceived as the absence of health, tends to be broader than that of disease. From the medical perspective, the term “disease” implies some aspect of delimitation and classification. This comes close to the definition in the Webster's dictionary [8], where disease is defined as "*a condition of the living animal or plant body or of one of its parts that impairs normal functioning and is typically manifested by distinguishing signs and symptoms*", whereas disorder is just an "*an abnormal physical or mental condition*". Therefore, it is mainly the correspondence to some kind of pre-defined pathophysiological pattern which constitutes the differentia for disease. The assumption that disease is something more specific than disorder also underlies the controversy whether certain behavioral conditions such as substance abuse are diseases or just disorders [9], which has its root in the so-called disease model of alcoholism [10].

We do not find any support for Scheuermann's categorial distinctions either if we look at the real-world usage of the terms "disorder" and "disease". A statistical analysis of literature abstracts in the whole MEDLINE corpus of the most frequent modifiers of the key words "disease" and "disorder" yielded the results presented in Table 1:

**Table 1:** Frequency of modifiers of the head words "disease" and "disorder" in MEDLINE abstracts

"disease" in MEDLINE		"disorder" in MEDLINE	
137,880	heart disease	22,360	bipolar disorder
77,167	artery disease	20,496	psychiatric disorders
66,710	cardiovascular disease	14,907	stress disorder
59,307	liver disease	14,458	depressive disorder
42,607	renal disease	14,115	anxiety disorders
34,857	pulmonary disease	13,977	mental disorders
29,143	kidney disease	13,935	personality disorder
27,999	bowel disease	13,600	panic disorder
27,927	lung disease	13,220	hyperactivity disorder
26,376	vascular disease	11,089	eating disorders

This analysis shows a clear preference of the word "disorder" with regard to abnormal behavior, whereas "disease" is the preferred word for organic abnormalities.

## Materials and methods

### Basic definitions

As a consequence of the above mentioned investigations we will abandon the terms *"disease"* and *"disorder"* in order to avoid language-specific connotations and rename them using the more neutral terms "*pathological structure*", "*pathological disposition*", and "*pathological process*", modifying the OGMS definitions as follows:

• Pathological structure:

*"a combination of bodily components that is causally linked to an elevated risk of pain or other feelings of illness, or dysfunction on the part of the organism, or of death; and that it is such that this elevated risk exceeds a certain threshold level*".

• Pathological disposition:

"*a disposition to undergo a pathological process that exists in an organism because of the presence of one or more pathological structures in that organism.*"

• Pathological process:

"*a bodily process that is causally linked to an elevated risk of pain or other feelings of illness, or dysfunction on the part of the organism, or of death; and that it is such that this elevated risk exceeds a certain threshold level". It may be the manifestation of a pathological disposition, located in a pathological structure, and have pathological structures as participants.*"

We have deliberately simplified these definitions. We do not address the highly controversial notion of a canonic life plan of an organism as in [4], as the boundary between normal and abnormal is not a topic to be discussed in this article. Furthermore we also allow for pathological processes that are not manifestations of pathological dispositions. We might even reconsider a renaming of these terms from "pathological" to "clinically relevant".

### The SDP representational framework

Along with many other ontology projects we use the Semantic Web standard OWL-DL [11] as a description logics [12] implementation because it has a well-understood semantics and is served by popular tools and classifiers, such as Protégé [13] and HermIT [14]. For the SNOMED CT redesign proposal we restrict ourselves to the less expressive OWL2-EL dialect that comes near to the formalism underlying the current release of SNOMED CT [5].

We have included this approach into the experimental upper level ontology BioTop [15,16]. BioTop provides basic categories and relations for health care and biomedical sciences and includes mappings to BFO [17], DOLCE [18], the OBO Relation Ontology [19], and the UMLS Semantic Network [20,21]. We use the description logics Manchester syntax ('subClassOf' for subsumption, 'equivalentTo' for equivalence, 'or' for disjunction, 'and' for conjunction, 'some' for existential restriction, 'only' for value restriction. 'not' for negation) [22]. We use *italic* font for class symbols and **bold face** for relation symbols.

Our approach is centered on the main categories *Pathological**S**tructure*, *Pathological**D**isposition*, and *Pathological**P**rocess*, hence "SDP". These three fundamental categories are placed as follows ("*bt*:" symbolizes the BioTop namespace):

*bt:PathologicalStructure* subClassOf *bt:MaterialEntity* subClassOf *bfo:IndependentContinuant*

*bt:PathologicalDisposition* subClassOf *bfo:Disposition* subClassOf *bfo:DependentContinuant*

*bt:PathologicalProcess* subClassOf *bt:ProcessualEntity* subClassOf *bfo:Occurrent*

All three categories are mutually disjoint:

*bt:PathologicalStructure* and *bt:PathologicalDisposition* subClassOf Nothing

*bt:PathologicalStructure* and *bt:PathologicalProcess* subClassOf Nothing

*bt:PathologicalProcess* and *bt:PathologicalDisposition* subClassOf Nothing

**Figure 1:**
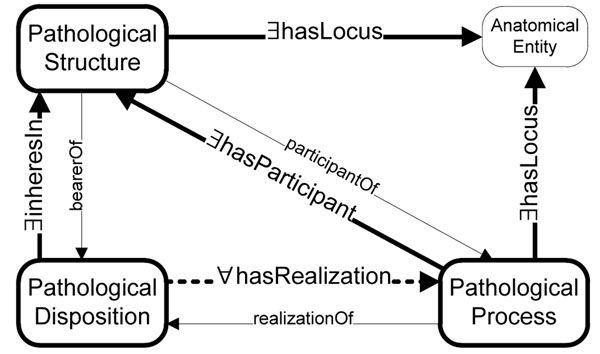
Relations between instances of the classes in the pathological structure–disposition–process triple. All classes and relations are in the namespace *bt:*. Thick continuous lines represent existentially quantified relations for all instances of the originating class (e.g. all instances of *PathologicalDisposition***inhereIn** SOME instance of *PathologicalStructure*). The dotted line stands for a universally quantified relation (value restriction) for all instances of *PathologicalDisposition* (e.g. all instances of *PathologicalDisposition*** hasRealization** ONLY in instances of class *PathologicalProcess* iff they have at least one relation of that type). The thin continuous lines represent relations that hold existentially for certain subclasses of these classes (e.g. all instances of the class *InfarctOfMyocardStructure***participantOf** SOME instance of *MyocardialInfarctionProcess*).

The relations between instances of these classes are depicted in Figure 1. Instances of *bt:PathologicalStructure*, as material entities (e.g. tumors), can be related to anatomical entities (e.g. organs, tissues, spaces) by the mereotopological relations **bt:physicalPartOf** and **bt:physicallyLocatedIn**, both subclasses of **bt:hasLocus**. They are furthermore related to instances of *bt:PathologicalProcess* by the relations **bt:participatesIn** and **bt:locusOf**. Instances of *bt:PathologicalProcess* can be related to their underlying dispositions by the relation **bt:realizationOf**. Instances of *bt:PathologicalDisposition* are related to instances of *bt:PathologicalStructure* by the relation **bt:bearerOf** (inverse **bt:inheresIn**). Ontological dependency can be stated both for pathological dispositions on pathological structures, and for processes on body structures, which are not necessarily pathologic:

*bt:PathologicalDisposition* subClassOf **bt:inheresIn** some *bt:PathologicalStructure*

*bt:PathologicalProcess* subClassOf **bt:hasParticipant** some *bt:BodyStructure*

In contradistinction to the Scheuermann approach [4] we do not claim that all pathological processes are manifestations of pathological dispositions. We therefore do not include the axiom:

* bt:PathologicalProcess subClassOf **bt:realizationOf** some *bt:PathologicalDisposition*

In contrast, we state:

*bt:PathologicalDisposition* subClassOf **bt:hasRealization** only *bt:PathologicalProcess*

By this and related axioms we express that dispositions of a kind, although being inherent in the things they are ascribed to, point to their realization in the future, which is only hypothetical. Once realized, they can only have a manifestation of a certain type.

Certain pathological processes are always realizations of certain dispositions. In these cases we formulate the constraint:

*PathologicalProcessX* subClassOf **bt:realizationOf** some *bt:PathologicalDispositionX*

### Examples

Some examples may illustrate these relationships and dependencies.

• A gene defect type *gStruct* is a child of *bt:PathologicalStructure* and all instances are **bt:bearerOf** a *bt:PathologicalDisposition* of the type *gDis*. These dispositions are only realized by processes of the type *gProc*. The classical example is Huntington's disease, caused by a defective allele on the fourth chromosome (4p16.3, see http://www.ncbi.nlm.nih.gov/omim/143100). Regardless the process leading to an initial mutation in the gene locus 4.16.3, the initial *bt:PathologicalStructure* is an expanded trinucleotide repeat (CAG)n, encoding glutamine, in the gene encoding the protein *Huntingtin* on chromosome 4.16.3. It is **bt:bearerOf** the *bt:Dispostion* to be transcribed and translated to *Huntingtin* with an expanded polyglutamine strand at the N terminus (It is debatable whether this translation process should be named pathological, which again puts into question the meaningfulness of the "pathological / non-pathological" dichotomy). This example of a genetically determined disease shows that for transcription and translation processes in the initial SDP triplet from the genetic structure (pathological information) into a pathological protein not necessarily *bt:PathologicalDisposition* and *bt:PathologicalProcess* are involved. The generation of a pathological protein as another *bt:PathologicalStructure* is the next step in a cascade of SDP triplets from molecular alteration, over cellular damage to morphological brain damage with clinical symptoms. Briefly, *Huntingtin* with expanded polyglutamine strand is **bt:bearerOf** the *bt:PathologicalDisposition* to aggregate and to interact with other proteins. In the following steps, aggregates and altered protein components as new instances of *bt:PathologicalStructure* induce impaired neuronal cell function and cell death. A variety of animal models are available to investigate subsequent steps in the pathogenesis of Huntington’s disease. The SDP representation of pathology distinguishes structure from process and allows an exact mapping to ontologies of biological structure, process or pathology.

• An *Allergy* is a *bt:PathologicalDisposition* of specific components of the immune system of an organism. All instances of the process type *AllergicReaction*, are realizations of a disposition of this type, and have an allergen as their (agentive) participant. In the case of *Allergic Rhinitis*, the disease process brings about a quality change in the nasal mucosa which exhibits signs of an inflammatory morphology. This pathologically altered anatomical structure is also referred to by clinicians as *Allergic Rhinitis*, so that in this case the ambiguity is not between the bearer of a disposition, the disposition itself and its realization as in the standard model, but between the disposition, the realization and a participating entity which undergoes a quality change.

• A specific binding of the chemical thalidomide to DNA forms a pathological structure at the molecular level which is **bt:bearerOf** the pathological disposition realized by the ensuing abnormal development of limbs. One possible result is the absence of both forearms, which can be ontologically described as a human body that has no parts of the type forearm. This final resulting structural malformation itself is not necessarily the bearer of pathological dispositions, so that no pathological process has to be expected. However, these organisms lack some of the dispositions (e.g. handwriting) that normally inhere in a well-formed organism.

• There are numerous other molecular structural defects bearing a disposition for abnormal development that themselves bear pathological dispositions. For instance, a structurally abnormal chromosome 21 is the bearer of a specific disposition that can be realized by a pathological process, *viz.* the development of a ventricular septal defect (VSD), a heart defect. The outcome of this process, i.e. the VSD itself, constitutes a pathological structure that is the bearer of another disposition, which can be realized by certain processes, such as pulmonary hypertension.

• A bacterial pneumonia starts with the proliferation of bacteria in a part of the lung and causes a pathological alteration of the tissue structure. This occurs only if the quantity or the pathogenicity of the damaging agent exceeds a certain threshold above which compensation by the organism is no longer possible. A preexisting pathological structure such as lung edema can alter this threshold by increasing the susceptibility for the proliferation of microorganisms — this constitutes, again, a disposition of that structure. The specific damage to lung tissue, as a pathological structure, bears several dispositions which can be manifested by new processes of characteristic tissue reactions that cause further pathological alterations such as consolidation.

• A similar example is given with a fracture. The fracture event is a nearly instantaneous process caused by an external force, which has a pathological structure as its characteristic outcome. This event is, however, not the realization of a pathological disposition. Normal bones have, of course, the disposition of breaking under extreme forces, but this disposition is not pathological. Only in the special case of a pathological fracture, i.e. a fracture of a pathologically fragile bone such as the result of osteoporosis, the event is the realization of a pathological disposition. A fractured bone has many pathological dispositions that can result in a variety of pathological processes, for example the development of a nonunion of fracture. However, it also has the disposition that is realized by a healing process, which is a second process but not a pathological one. Often, the term "fracture" is indiscriminately used both to denote the traumatic event and the healing phase, or just the broken bone with or without surrounding soft tissue damage.

These examples suggest that instances of *PathologicalStructure*, *PathologicalDisposition*, and *PathologicalProcess* can be identified in most diseases. However, in many cases it is difficult to ontologically clearly “detect” initial pathological structures due to chemical, physical, or biological agents in a sequential chain of structure-disposition-process iterations. On the one hand, threshold effects play an important role as long as minor alterations are physiologically compensated, so that it remains difficult to delineate the threshold of pathological damages. Furthermore, there are cases in which two clearly distinct processes must be distinguished, e.g. trauma and healing. If we consider the traumatic event separately, we usually have no pathological dispositions. There are pathological processes during which new pathological structures come into being, but it is also noteworthy that not all processes in an SDP chain are generically pathologic. In addition, not all pathological structures are bearers of new pathological dispositions, the manifestation of which would then be complications of the previous pathological process. Finally, the ambiguity inherent in naming something a disease not always comprises the classical SDP entities, as there are cases (e.g. allergies) in which the same name is used for a disposition, its manifestation, and a structural outcome of this manifestation.

## Results

### The BioTop solution

Ontology engineering should be guided by parsimony and scalability. This means on the one hand keeping the ontology as simple as possible provided it meets the requirements for knowledge representation, and on the other hand adding details, expressivity, and complexity in a non-disruptive way. With regard to the representation of diseases, this means that a naïve model which ignores the structure/disposition/process distinction should be made up in a way that allows a "graceful evolution" [23] towards a more sophisticated ontology.

One result of our investigation is a decision to conflate the three categories, which can be straightforwardly expressed by the new BioTop category *bt:PathologicalEntity*, defined by disjunction:

*bt:PathologicalEntity* equivalentTo* bt:PathologicalStructure* or *bt:PathologicalDisposition* or *bt:PathologicalProcess*

A common feature of all instances of subclasses of the *bt:PathologicalEntity* class is that they are linked to some anatomical entity. In formalizing this we encounter the following difficulties:

• All instances of *bt:PathologicalStructure* are related to the anatomical objects where they occur via the relation **bt:physicalPartOf** or by the more general relation **bt:physicallyLocatedIn**.

• All instances of *bt:PathologicalDisposition* are related to their bearers by the relation **bt:inheresIn**.

• All instances of *bt:PathologicalProcess* are related to the place where they occur by the generalized localization relation **bt:hasLocus**, and to their participating entities by **bt:hasParticipant**.

BioTop exhibits the following relation hierarchy:

**bt:physicalPartOf** subClassOf **bt:physicallyLocatedIn** subClassOf **bt:hasLocus**.

We will now extend the scope of the transitive relation **bt:hasLocus** so that it is also a parent relation of **bt:inheresIn**. That means that whenever a disposition of the type *D* inheres in some structure of the type *S* then it is also necessarily located in *S*.

If *S* is part of *S′* then *D* is also located in *S*′:

*D* subClassOf **bt:inheresIn** some *S* entails *D* subClassOf **bt:hasLocus** some *S*

*S* subClassOf **bt:physicalPartOf** some *S*′ entails 	*S* subClassOf **bt:hasLocus** some *S*′

*D* subClassOf **bt:inheresIn** some S entails *D* subClassOf **bt:hasLocus** some *S*′

Already before the redefinition of the relation **bt:hasLocus** we were able to relate both pathological structures and processes to anatomical entities by the relation **bt:hasLocus**. A tumor of the sigmoid colon can therefore be classified as a tumor of the colon and as a tumor of the intestine regardless of whether we consider the referent of the term "tumor" to be in the category *bt:PathologicalProcess* or *bt:PathologicalStructure*. After the redefinition we have a uniform way of linking dispositions, too, in the same way. As an example, the defect of a gene allele which resides on a chromosome can related to the pertaining chromosome class by **bt:hasLocus**, regardless whether it is seen as a structure or a disposition.

This so-called propagation of attributes [24,25] via partitive or locative relationships ("a disease located in a part is also located in the whole") is considered primordial for reasoning in biomedical ontologies. We should therefore ask whether the related reasoning patterns are guaranteed even in an ontology that deliberately refrains from the distinction between pathological structures, processes and dispositions. Processes are related to spatial or material entities in different ways. On the one hand they have a general location, which expands transitively; e.g. pneumonia processes are in the lung and therefore in the body, but not in the liver, as the latter does not spatially overlap with the lung. On the other hand they have participants as their causal agents, which come into or transition out of being during the process, or undergo structural changes. Process participants behave differently compared to process locations, as they do not propagate from parts to wholes – a gangrene *of* the toe is not a gangrene *of* the foot, but a gangrene *located in* a toe is a gangrene *located in* a foot. The use of the very global relation **bt:hasLocus**, which includes the notion of inherence of a disposition, the location of a process and the relation of anatomical parts to wholes, guarantees this reasoning pattern even for non-committed subclasses of *bt:PathologicalEntity*.

Only processual entities have participants. Therefore, if an ontology engineer refines a class *P* in the *bt:PathologicalEntity* branch by a **bt:hasParticipant** role, a classifier infers that *P* must be a subclass of *bt:PathologicalProcess* and therefore, disjoint from material or dispositional entities. If some *P* has both a **bt:hasParticipant** role and a **bt:inheresIn** role, *P* is unsatisfiable, because of the domain constraints **bt:hasParticipant** some *Thing* subClassOf *bt:ProcessualEntity* , and **bt:inheresIn** some *Thing* subClassOf not *bt:ProcessualEntity*.

In an ontology engineering process this would be the signal that two distinct classes for the pathological disposition and its realization need to be created. An example is the gene defect for Huntington's disease and its manifestation, which has other participants than just the ill-structured gene, or the distinction between the allergic disposition and the allergic process.

### Adaptation to SNOMED CT

The confusion between pathological structure, disposition, and process is widespread in SNOMED CT, currently the largest ontology project in the biomedical domain [5].

All representational units in SNOMED CT are named "SNOMED CT concept". In this example we interpret SNOMED CT "linkage concepts" (such as **sct:AssociatedMorphology**) as OWL object properties and all others as OWL classes. "sct" is the identifier of the SNOMED CT namespace.

Briefly, SNOMED CT distinguishes, on its upper level, the categories of *sct:BodyStructure* (30,619 concepts) and *sct:ClinicalFinding* (97,139 concepts). A subclass of *sct:BodyStructure* is *sct:MorphologicAbnormality* (4,335 concepts), which encompasses all sorts of morphological alterations. The *sct:ClinicalFinding* hierarchy exhibits a more specific subdivision *sct:Disease* (64,161 concepts). Upon ontological scrutiny, numerous classes in this hierarchy are ambiguous with regard to their interpretation as either processes or dispositions. Furthermore, there are finding classes that simply restate the existence of some morphologic abnormality such as *sct:ColostomyPresent*. Finally, in a separate hierarchy named *sct:Event* (3,656 concepts) we find classes like *sct:Asphyxiation* and *sct:Suicide* both of which can also be encompassed by a broad understanding of "Disease".

The SNOMED CT relationship **sct:FindingSite** relates finding and disease classes with canonical anatomic entities where they are located or which are involved, whereas the relation **sct:AssociatedMorphology** is used to relate them with non-canonic structures.

There is no relation that relates morphology classes with canonic anatomy classes. The main reason for this is that the morphology hierarchy is restricted to very general morphological structure classes which are not refined in terms of specific anatomical sites. These more specific classes are generally found in the finding hierarchy. For instance, *sct:FibrosisOfPleura* implies the expression

(**sct:AssociatedMorphology** some *sct:Fibrosis*) and (**sct:FindingSite** some *sct:PleuralMembraneStructure*)

*sct:FibrosisOfPleura* is therefore not a subclass of *sct:Fibrosis*.

In order to apply the SDP approach to SNOMED CT we first analyze the relations involved.

**sct:FindingSite** can be mapped to the BioTop relation **bt:hasLocus**. However, specialized location relations of dispositions, processes, and structures cannot be differentiated by relation refinement in SNOMED CT analogously to BioTop because of missing sub-relations of *sct:FindingSite*.

Therefore, we suggest introducing the possibility to differentiate between dispositions and processes by introducing two new top level classes *sct:PathologicalDisposition* and *sct:PathologicalProcess* in the *sct:Finding* hierarchy of SNOMED CT which can be used as parents of ambiguous concepts.

In the case of the ambiguous class *sct:AllergicRhinitis* it is now possible to create two new subclasses for the disposition and its realization and fully define them as descendants of either *sct:PathologicalDisposition* or *sct:PathologicalProcess*:

*sct:ManifestAllergicRhinitis* equivalentTo *sct:AllergicRhinitis* and *sct:PathologicalProcess*

*sct:AllergicRhinitisDisposition* equivalentTo *sct:AllergicRhinitis* and *sct:PathologicalDisposition*

For a visualization see Figure 1: the class D2 of the current SNOMED CT hierarchy, shown on the left side, has two subclasses D2a (of *PathologicalDisposition*) and D2b (*PathologicalProcess*) in the redesigned hierarchy, as shown on the right side.

A differentiation between process and structure could be done in a similar way:

*sct:PleuralFibroticProcess* equivalentTo *sct:FibrosisOfPleura* and *sct:PathologicalProcess*

*sct:PleuralFibroticStructure* equivalentTo *sct:FibrosisOfPleura* and *sct:PathologicalStructure*

In the latter case, however, we encounter the problem that the proposed *sct:PathologicalStructure* class corresponds to *sct:MorphologicAbnormality* and that therefore the resulting expression *sct:PleuralFibroticStructure* would correspond to

(**sct:AssociatedMorphology** some *sct:Fibrosis*) and (**sct:FindingSite** some *sct:PleuralMembraneStructure*) and *sct:MorphologicAbnormality*

from which the subsumption

*sct: PleuralFibroticStructure* subClassOf *sct:Fibrosis*

cannot be drawn.

The morphology-disease dichotomy in SNOMED CT enforces the view that terms that could alternatively be interpreted as denoting structures or processes, necessarily denote abnormal structures on a more abstract level (e.g. *sct:Fibrosis*) and processes or dispositions on a more specific level (e.g. *sct:FibrosisOfPleura*). If we want to represent *sct:PleuralFibroticStructure* we need to use the postcoordinated expression *sct:Fibrosis* and *sct:PleuralMembraneStructure*. If, on the contrary, we want to represent *sct:FibroticProcess* we can postcoordinate it as *sct:PathologicalProcess* and (**sct:hasMorphology** some *sct:Fibrosis*).

**Figure 2:**
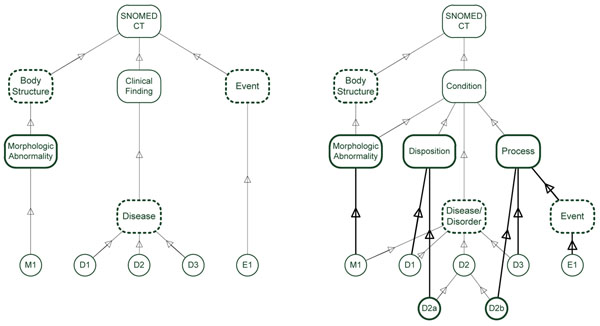
SNOMED CT redesign according to the SDP modeling approach. Peculiar SNOMED CT concepts are encased in dotted boxes to be better identifiable in the new model. The concepts of the structure – disposition – process triple and the resulting subsumptions to them are emphasized by bold lines. *Left:* Current SNOMED CT hierarchy (simplified). Diseases do not distinguish between pathological processes and pathological disorders. Morphological abnormalities are in a separate hierarchy, as well as events. *Right:* Redesigned upper level. Clinically relevant structural abnormalities, dispositions and processes are subsumed by a new "Condition" node. The former disease classes can be disambiguated by introducing a new is-a link to *Process* and *Disposition*, respectively (example D2a and D2b). Morphological abnormalities are also subsumed by *Disease*/*Disorder*. Events are processes.

Fig. 2 shows the proposed redesigned structure of SNOMED CT, with a new, disjunctive class *sct:Condition*, the SNOMED CT correspondent of *bt:PathologicalEntity*, which encompasses all kinds of clinical relevant phenomena. The node *Disease*/*Disorder* is broader and encompasses also pathological structures.

The scalable approach we propose can easily be used for refining ambiguous SNOMED CT disease and/or finding concepts in terms of processes or dispositions. It cannot, however, be applied to distinguish structures. SNOMED CT has already a strong ontological commitment in the sense that more general terms are meant as structures and more specific ones as non-structures, at least if we assume the finding and the body structure hierarchies as disjoint. This is further underlined by debatable duplications of completely disconnected SNOMED CT concepts such as *sct:BlastCell* in the body structure hierarchy and *sct:BlastCellsPresent* in the finding hierarchy.

## Conclusion

The ontological triad structure – disposition – process (SDP) is suited to describe iterations of sequential complex pathological processes in which the outcome of one pathological process is a pathological structure that bears a pathological disposition, which may be realized in a subsequent step by a specific pathological process. Many applications, however, are well served with a much less complex approach, in which the above distinction is less relevant. By introducing the disjunctive class *bt:PathologicalEntity* (*sct:Condition*) we are able to represent diseases without specifying the ontological category, and to relate them to anatomical objects and spaces via the **bt:hasLocus** relation. This approach allows the non-disruptive, graceful evolution towards more sophisticated representations. In a case study we have investigated how the proposed approach can be used in the current redesign of events, conditions, and episodes in SNOMED CT. Here we found that numerous disease and finding concepts are indeed ambiguous and can be interpreted either as processes or as dispositions. By introducing the SDP top level, disambiguation, where necessary, is straightforward. For the sake of maintaining ontologies simple and introducing more complex structures only where required, we defend a judicious use of disjunctive, and therefore ambiguous, classes in biomedical ontologies during the process of ontology construction and in the practice of application provided the intended reasoning scenarios are supported.

## Authors' contributions

SS conceived and formalized the proposed ontology design pattern. SS and MB sketched the use cases. KS, AJ, and CC added SNOMED CT related aspects. All authors helped to draft the manuscript. They read and approved the final paper.

## Competing interests

'The authors declare that they have no competing interests.
